# Inhibition of in-stent restenosis after graphene oxide double-layer drug coating with good biocompatibility

**DOI:** 10.1093/rb/rbz010

**Published:** 2019-03-19

**Authors:** Shuang Ge, Yadong Xi, Ruolin Du, Yuzhen Ren, Zichen Xu, Youhua Tan, Yazhou Wang, Tieying Yin, Guixue Wang

**Affiliations:** 1 Key Laboratory for Biorheological Science and Technology of Ministry of Education, State and Local Joint Engineering Laboratory for Vascular Implants, Bioengineering College of Chongqing University, Chongqing, China; 2 Department of Biomedical Engineering, The Hong Kong Polytechnic University, Hong Kong SAR, China

**Keywords:** graphene oxide, drug coating, vascular stents, restenosis, thrombosis

## Abstract

In this study, we designed a double layer-coated vascular stent of 316L stainless steel using an ultrasonic spray system to achieve both antiproliferation and antithrombosis. The coating included an inner layer of graphene oxide (GO) loaded with docetaxel (DTX) and an outer layer of carboxymethyl chitosan (CMC) loaded with heparin (Hep). The coated surface was uniform without aggregation and shedding phenomena before and after stent expanded. The coating treatment was able to inhibit the adhesion and activation of platelets and the proliferation and migration of smooth muscle cells, indicating the excellent biocompatibility and antiproliferation ability. The toxicity tests showed that the GO/DTX and CMC/Hep coating did not cause deformity and organ abnormalities in zebrafish under stereomicroscope. The stents with GO double-layer coating were safe and could effectively prevent thrombosis and in-stent restenosis after the implantation into rabbit carotid arteries for 4–12 weeks.

## Introduction 

The accelerated aging of the population has led to the continuous increase in the incidence of coronary artery disease (CAD). The World Health Organization predicts that the total number of CAD-induced deaths will rise to 24 million by 2030 worldwide [1, 2]. Percutaneous coronary intervention, such as stent implantation, has become the most important and effective technique for the diagnosis and treatment of cardiovascular diseases [3]. However, the implantation of vascular stents usually causes endothelial injury and exfoliation, leading to the late thrombosis and restenosis [4]. Drug-eluting stent (DES) can effectively prevent the in-stent restenosis (ISR) but inhibit the proliferation of endothelial cells due to the drug release during the early stage, resulting in high local drug concentration [5]. Therefore, a novel strategy is desirable to ensure drug loading and the effective release.

Graphene oxide (GO), as a 1D structure of graphite, has a large specific surface area [6]. The rich functional groups in GO enable it to become a flexible platform for post-modification with excellent adsorption capacity through both physical adsorption and covalent interaction with drugs carrying negative charge [7, 8]. Therefore, GO has been widely used as a drug carrier in the field of biomedical engineering [9]. Barahuie *et al*. [10] prepared GO through a modified hummer process, which was loaded with active anticancer drug chlorogenic acid (CA). The loading of CA in the nano-mixture was ∼13.1%. Kim *et al*. combined reduced GO with polyethylene glycol (PEG) and found that PEG–BPEI–rGO could load more doxorubicin via π–π bond and hydrophobic interaction than unreduced GO and PEG [11]. Besides serving as drug carriers, GO has also been involved in cardiovascular and other fields. Jing *et al*. [12] prepared a thermoplastic polyurethane–GO small-caliber artificial blood vessel by electrospinning, which could promote cell adhesion and growth on the surface. These results may open up a new direction for GO in the treatment of cardiovascular diseases. Nevertheless, the applications of GO in this area remain limited.

In this study, we designed the stents coated with a new type of GO double layer to achieve anti-proliferation and anti-thrombosis. Dopamine (DA) was used to modify 316L stainless steel (316L SS) stents, in which the inner layer was coated with GO loaded with docetaxel (DTX) and the outer layer was coated with carboxymethyl chitosan (CMC) loaded with heparin (Hep) [13, 14]. A series of *in vitro* and *in vivo* experiments were carried out to verify its effectiveness and biosafety.

## Materials and methods

### Materials

316L SS stents that provided by Northwest Institute for Non-ferrous Metal Research (Xian, China).

Twelve New Zealand white rabbits (male, 2.5–3.5 kg) were purchased from Chongqing Medical University. All animals were randomly divided into two groups, GCC stents (*n* = 6) and 316L SS stents (*n* = 6). Three days prior to surgery, aspirin (2 mg/kg) and clopidogrel (1.5 mg/kg) were added to the normal diet. Chongqing Medical University ethics review board granted animal ethics approval.

### Preparation of GO double layer drug coating (GO/DTX+ CMC/Hep coating, GCC)

316L SS stents polished by different specifications of sandpaper and mirror polished by 0.3 and 0.5 μM alumina polishing liquid successively. Then all stents were dehydrated at 40°C for 8 h in vacuum oven after ultrasonic cleaning (10 min, respectively, with 75% ethanol, acetone and distilled water).

**Figure 1:**
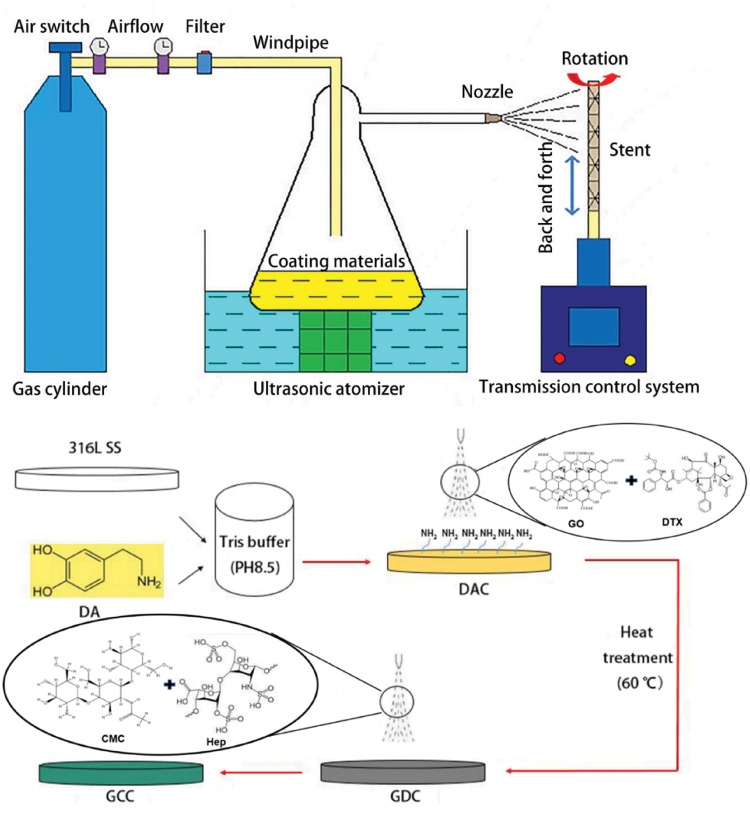
The coating processing and the CS-III pro-ultrasonic spray system [15]. DAC, dopamine coating; GDC, DAC+GO/DTX; GCC, GDC+CMC/Hep

The stent-spraying system and coating steps was shown in Fig. 1 [15, 16]. The bare metal stents were immersed in the 2 mg/ml DA solution (pH 8.5 Tris Buffer) for 12 h. DTX was dissolved in GO solution (2 mg/ml). Hep and CMC dissolved in tri-distilled water. Stents and sheets coating were prepared by the vascular stent ultrasonic atomization micro-drug sprayer CS-III pro (setting the parameters: process engineering number was 25, stent spin velocity was 9 r/s and forward velocity was 4 r/s) which was developed in previous researches [17].

### Characterization of different coatings

The morphology changes of each coating (316L SS group, DAC group, GDC group, GCC group) were characterized by scanning electron microscopy (SEM) from 15 to 30 kV (Vega-LMH, Tescan, Czech). The roughness value (Ra) of different coating was measured by atomic force microscope (AFM; MPF-3D-BIO, Asylum Research, USA) own software MFP3D 13.11.94. The hydrophilicity alteration of different coating was measured with a contact angle tester (DSA30, Krüss, Germany) at room temperature.

### *In vitro* hemolysis test

Hemolysis ratio was carried out following the Chinese national standard (Biological Evaluation of Medical Devices-Part 4: Selection of Tests for Interactions with Blood, *GB/T 16886.4-2003*) [18]. Four different groups of stents were static pre-incubated in 10 ml of normal saline for 30 min at 37°C, and then 0.2 ml of diluted anticoagulated blood sample was added for another 60 min.

### Platelets adhesion test

All samples were incubated in 0.5 ml of platelet-rich plasma for 30 min at 37°C, then fixed and dehydrated for SEM. The deformability of adherent platelets (types I–V) was analysed randomly per 1000 × field (*n* = 5), and the Platelet morphology index (DI) was calculated via the following formula [19]:

(1) $$\mathrm{DI}\;=\frac{（\mathrm{No}.\;\mathrm{type}\;I\times 1\;+\;\mathrm{No}.\;\;\mathrm{type}\;\mathrm{II}\times 2+\mathrm{No}.\mathrm{type}\;\mathrm{III}\times 3+\mathrm{No}.\mathrm{type}\;\mathrm{IV}\times 4+\mathrm{No}.\;\mathrm{type}\;V\;\times \;5）}{\mathrm{Total}\;\mathrm{number}\;\mathrm{of}\;\mathrm{adherent}\;\mathrm{platelets}}$$

### Vascular smooth muscle cells proliferation experiments

Vascular smooth muscle cells (VSMCs) seeding of the stents was carried out in rotation culture equipment according to our previous studies [20]. The growth and adhesion of VSMCs on the stents were monitored with SEM. Finally, the 490 nm OD values were measured by Multiskan Spectrum (Thermo Scientific, USA) (*n* = 3).

### *In vivo* zebrafish toxicity tests and bio-safety evaluation of GCC stents

#### Zebrafish toxicity tests of GCC

Flk-1 zebrafish (supplied by Prof. Anming Meng, Tsinghua University, Beijing, China) were maintained at a temperature of 28 ± 0.5°C under a 14 h:10 h light–dark cycle. Fertilized eggs were collected within 4 hour post-fertilization (hpf). For experiments, embryos were selected under stereomicroscope to ensure they were healthy and were in the same developmental stage. Thirty of the selected embryos were randomly placed in 24-well culture plates with triplicates for each group. The survival rate, hatching rate and heart rate at 24, 48 and 72 hpf were calculated. Ten zebrafish embryos or juvenile fish were randomly selected from the previous groups and observed under light microscopy for embryonic development at 24, 48 and 72 hpf.

#### Bio-safety evaluation after stent implantation to rabbits

Main organs such as myocardium, liver, spleen, lung and kidney of all rabbits were collected and hematoxylin–eosin (H&E) stained to observe the pathological structure. In addition, blood samples were taken from the central ear arteries for routine blood examination and serum biochemical examination.

#### *In vivo* animal experimental study of GCC stents

The animals were anesthetized by chloral hydrate (3.5 kg/ml) intraperitoneal injection. The stents were implanted into the left common carotid artery at a balloon pressure of 10 atm for 30 s. The deployment conditions are less than 1.2:1 (stent:artery) ratio. Animals were sacrificed at 1 and 3 months, and half of stented arteries were fixed with 2.5% glutaraldehyde for SEM examination.

The other half arteries with stents were sliced, then stained by H&E used for stenosis analysis after the stents were completely electrolyzed [15, 21].

### Statistical analysis

In this study, the data obtained were reported as means ±SD. Data obtained from different treatment groups were statistically compared by statistical software SPSS 11.5 (Chicago, USA). To reveal differences among the groups, one-way ANOVA followed by Tukey’s test was used. Differences were considered significant at *P < 0.05 and highly significant at **P < 0.01.

## Results

### Characterization of different coatings

**Figure 2:**
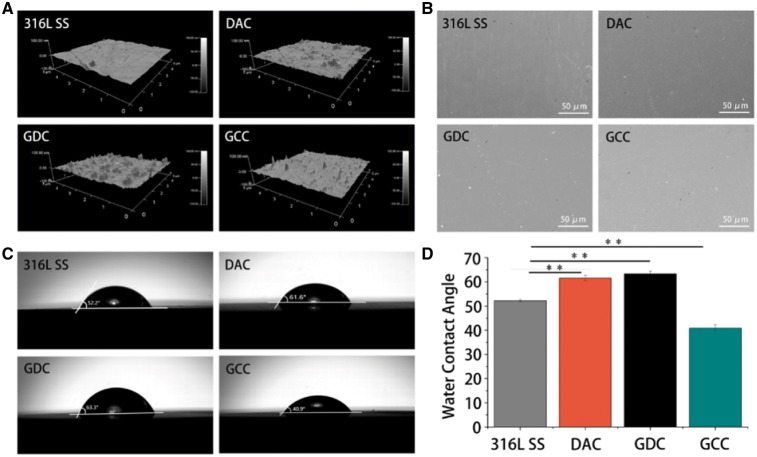
GCC coating has minimum effects on surface roughness but significantly decreases water contact angle. (**A**) The surface roughness after various coatings. 316L SS stents were coated with DAC, GDC or GCC and then imaged by atomic force microscopy. (**B**) Different surfaces were imaged by SEM. (**C**, **D**) GCC coating significantly decreases the water contact angle. **P < 0.01

Atomic force microscopy (AFM) was used to measure the surface morphology. Our data show that the 316L SS stent surface was smooth with the surface roughness Ra =2.203 ± 0.43 nm after the acid pickling pretreatment. However, the surface roughness significantly increased to 3.143 ± 0.81, 4.623 ± 1.10 and 2.771 ± 0.77 nm after DAC, GDC and GCC coating, respectively (Fig. 2A). The largest roughness in GDC might be related to the dispersion of GO in water. The roughness of DAC was larger than that of 316L SS and GCC, probably due to its non-controllable deposition and ultrasonic oscillation of the small shock and surface inhomogeneity. After coating with GCC, the roughness was reduced, which might be related to the water solubility of GCC. Furthermore, the SEM imaging results show that the surface of DAC, GDC and GCC was very uniform and no coating aggregation and did not show any significant difference (Fig. 2B).

Changes in surface roughness may alter the hydrophilicity, which then mediates the variations of absorbed proteins and influences the biocompatibility of the material. To evaluate the surface hydrophilicity after various coatings, water contact angle was used to measure the wettability of the material surface (Fig. 2C). The results show that the contact angle of 316L SS surface was 52.2 ± 0.51° and increased to 61.6 ± 1.10° and 63.3 ± 1.09° after the DAC and GDC coating, respectively (Fig. 2D). The contact angle after the GCC coating was decreased to 40.9 ± 1.32° and significantly lower than that of 316L SS, DAC, and GDC groups. Nevertheless, the contact angles were <90° in all groups, indicating the hydrophilic property of the material surfaces.

### Blood compatibility testing of different coatings

**Figure 3:**
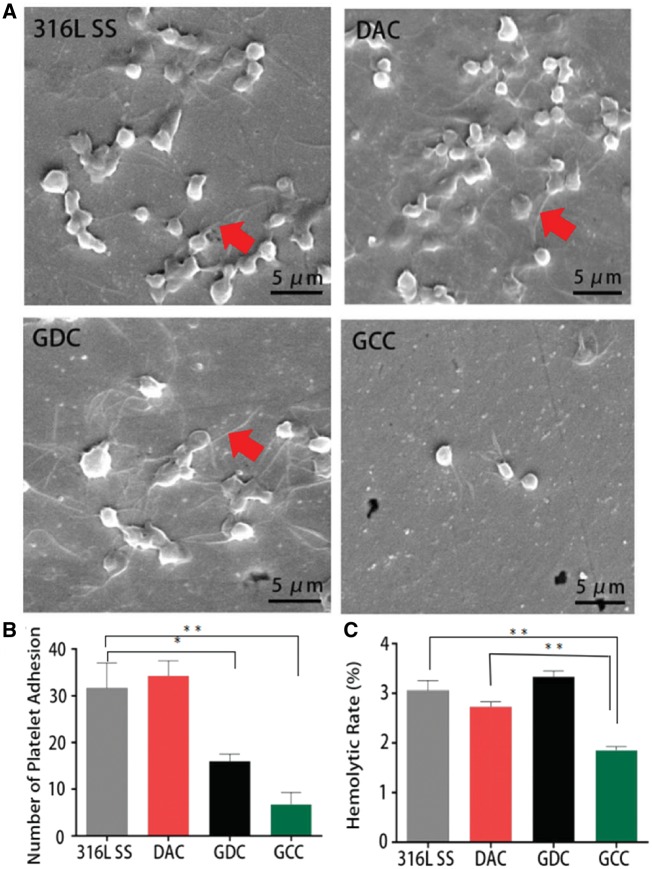
GCC Coating exhibits superior blood compatibility. (**A**, **B**) GCC coating inhibits the adhesion and activation of platelets. Platelets were plated on 316L SS surfaces with DAC, GDC or GCC for 1 h. The adhered cells were then fixed and imaged by SEM. Rectangular indicates cell spreading and aggregation, while red arrow represents pseudopods. The number of adhered platelets was quantified in (B). the level of platelet activation on the GCC surface (represented by the pseudopodia) was much lower than other three groups, while the GDC coating showed the highest platelet activation. (**C**) GCC coating exhibits low hemolysis rate. *P < 0.05; **P < 0.01

It is known that platelet morphology is related to its activation state [22]. To examine the activity of platelets adhered to the surfaces after various coatings, the adhesion and morphology of the adhered platelets were characterized by SEM. The platelets adhered to the GDC surface were less than that on 316L SS and DAC surfaces (Fig. 3A and B). The platelets adhered to the 316L SS, DAC and GDC surfaces exhibited obvious pseudopodia (Fig. 3A), suggesting that these platelets might be activated. Fewer platelets were found to adhere to the GCC surface and appear to be spherical and inactivated. Further analysis of platelet type showed that most of the platelets adhered to the 316L SS (21.6%) and DAC (36.2%) surfaces were type III (Table 1), while the largest portions of the platelets adhered to the GDC (37.2%) and GCC (40%) surfaces were type II and I, respectively. The morphology index of platelets progressively decreased from DAC (2.77), 316L SS (2.60), GDC (2.40) to GCC (1.93) surfaces, suggesting the continuous attenuation of platelet activation.

**Table 1:** Amounts and deformation index of different types of platelets on the surfaces with different coatings

Coating	Percentage of every type of platelet adhered to the surfaces (%)					DI
	I	II	III	IV	V	
316LSS	20.3	13.8	21.6	17.7	7.6	2.60
DAC	11.6	27.5	36.2	21.7	2.9	2.77
GDC	18.6	37.2	30.2	14.0	0	2.40
GCC	40	33.3	20	6.7	0	1.93

In addition, the hemolysis rates of the 316L SS stent, DAC, GDC and GCC were all below 5%, meeting the hemolysis test requirement according to the national biological safety guideline [23]. The hemolysis rates of 316L SS stent and GDC were significantly higher than that of DAC and GCC, among which the hemolysis rate of GCC was the lowest (Fig. 3C). There was no significant difference between 316L SS stent and GDC.

### The proliferation of VSMCs on different coatings

**Figure 4:**
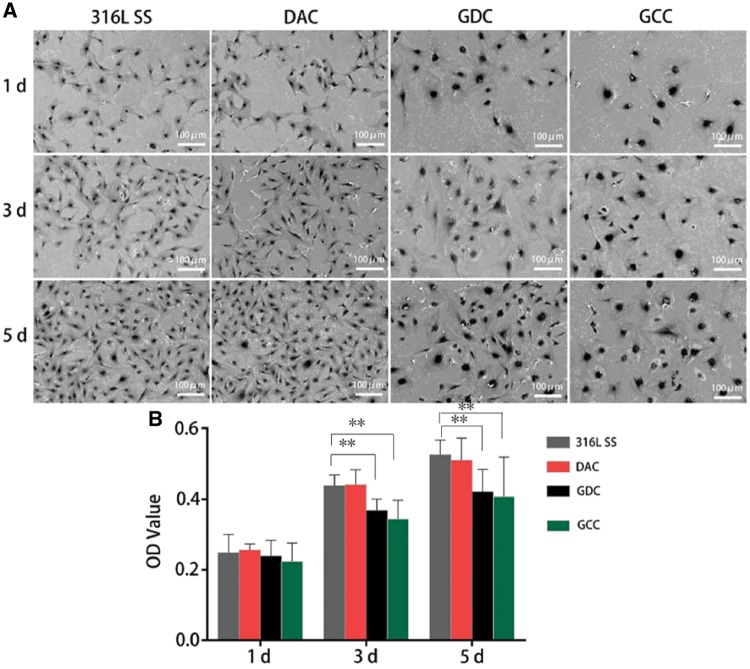
GDC and GCC Significantly inhibit the proliferation of VSMCs. VSMCs were plated on 316L SS coated with DAC, GDC or GCC. The images were taken at Days 1, 3 and 5, respectively (**A**). cell proliferation was measured by SEM at various time points accordingly (**B**). *n* = 3 independent experiments. **P < 0.01

The stents coated with drugs that inhibit VSMC proliferation are the most direct and effective ways to suppress ISR [24]. To explore the effects of various coating methods, the proliferation of VSMCs was measured on different coated surfaces. The results show that the coating with DTX (GDC and GCC groups) but not dopamine (DAC) notably inhibited the proliferation of VSMCs (Fig. 4A and B). Further addition of Hep (GCC) did not significantly inhibited VSMCs proliferation after co-culture for 3 and 5 days. In addition, our data show that GDC and GCC coatings not only inhibited VSMC proliferation but also affected cell morphology (Fig. 4A). The VSMCs adhered to GDC and GCC surfaces exhibited altered nuclear morphology and remodeled cytoskeletal structure, probably due to the effect of DTX on microtubules. These data suggest that coating stents with DTX may have the suppressive effects on VSMC proliferation and ISR.

### *In vivo* evaluation of stent toxicity and bio-safety

**Figure 5:**
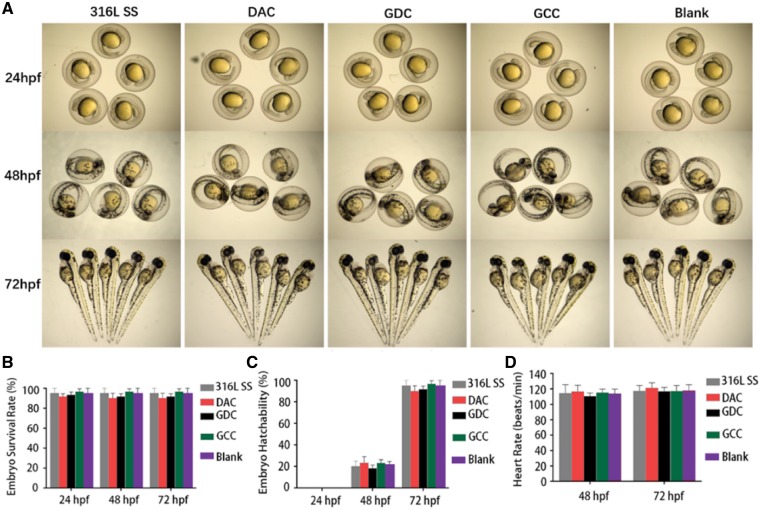
Various coatings exhibit no detectable toxicity in the embryonic development of zebrafish. Zebrafish embryos were co-cultured with 316L SS, DAC, GDC and GCC stents for 24, 48 and 72 h, respectively. The morphology (**A**), survival rate (**B**), hatching rate (**C**) and heart rate (**D**) of these embryos were measured at various time points. No significant difference was found among different groups, *n* = 3

Toxicity and bio-safety are crucial properties for stent implantation [25]. The toxicity of the stents with various coatings was further tested in zebrafish model system. Zebrafish embryos were co-cultured with the stents with differential coatings within 72 hpf. The results show that fertilized eggs exhibited more round morphology at 24 and 48 hpf in 316L SS, DAC, GDC and GCC groups but no distortion in the outer membrane and the embryonic development in the ovalbumin (Fig. 5A). No significant delay was found in the development of otoliths, eyes and hearts. The results of embryos at 72 hpf show that there was no significant difference in body length and size with excellent morphological development after incubating juveniles with various coatings (Fig. 5A). Furthermore, as the landmark indicators for zebrafish embryonic development, the survival rate, hatchability and heart rate were examined. Our data show that the survival rates were between 90 and 95% at 24, 48 and 72 hpf and there was no significant difference among different groups (Fig. 5B). No embryo was hatched before 48 hpf and the hatchability reached at 72 hpf in all groups (Fig. 5C), which is a common phenomenon in zebrafish development. There was no significant difference in hatchability among different groups. In addition, no difference was found in the heart rate of zebrafish embryos at 48 and 72 hpf (Fig. 5D). All these findings suggest that these coatings are not toxic to zebrafish embryonic development.

**Figure 6:**
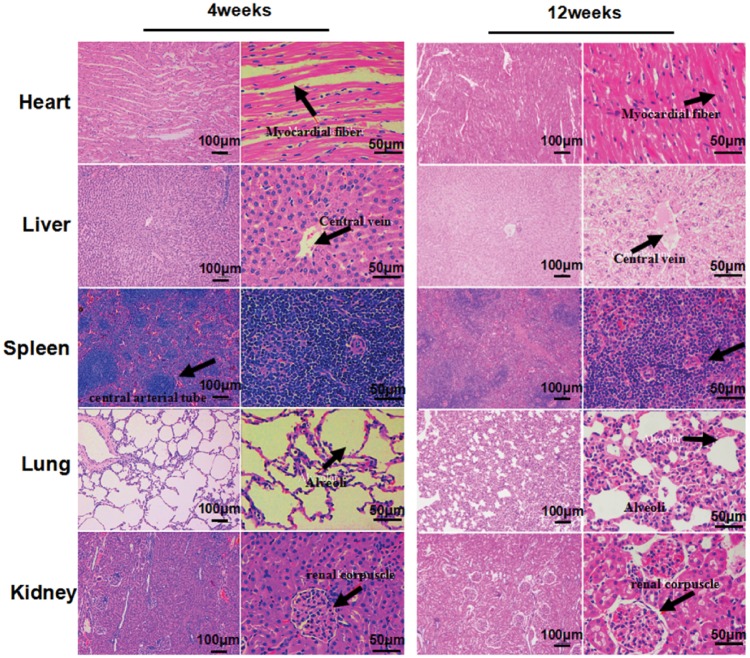
The GCC Stents have no significance effects on the main organs of rabbits at 4 and 12 weeks after implantation. The black arrows represent myocardial fiber, Central vein, Central arterial tube, alveoli and renal corpuscle

To characterize the bio-safety, 12 GCC stents were implanted successfully into 12 rabbits. These animals were then revived and fed regularly in the breeding center. During the experimental period (12 weeks), there were no significant differences between the animals with or without stent implantation in the physiological indices, such as diet and weight, also the indicators of routine blood examination and serum biochemical examination (Tables 2 and 3). Furthermore, no obvious differences were found in the color, luster and hardness of main organs such as heart, liver, spleen, lung and kidney. The results of H&E staining show no detectable differences in the structure and organization of these major organs (Fig. 6). Myocardial was structural integrity, myocardial fibers arranged closely, exhibited ladder pattern. No swelling or degeneration of hepatocytes and granules, hepatocyte cords were clear, and no thickening of central vein and hepatic sinus. The vascular structure of the central artery in white pulp of spleen was normal, and no thickening of the vascular wall was observed. The pulmonary alveolar contour was clear and identifiable, structure of alveolar wall remained integrity, and peripheral capillaries showed no abnormal enlarged phenomena. In the renal corpuscle structure, the glomerular morphology was plumpness, endothelial cells of glomerular and peripheral capillary were arranged regularly, and the morphological structure was plumpness. Renal vesicles and tubules showed no abnormalities. Therefore, the main organs in the experimental group had complete structure and no abnormal indexes. These findings suggest that the stent implantation is safe and non-toxic.

**Table 2:** The examination of blood biochemical indexes pre- and post-GO double layer drug coating stent implantation (*n*>3)

Project unit	Pre-stent implantation	Post-stent implantation
ALT (U/l)	49.47±17.42	47.45±18.87
AST (U/l)	17.15±3.69	29.32±10.47
TP (g/l)	55.528±2.41	53.98±3.24
ALB (g/l)	45.849±1.75	43.999±1.92
GLB (g/l)	9.779±1.78	9.981±2.62
TBIL (μmol/l)	0.127±0.11	0.256±0.07
ALP (U/l)	61.48±14.30	68.86±37.65
UREA (mmol/l)	8.726±1.97	7.497±1.58
CREA (mmol/l)	116.01±21.03	97.58±20.80
GLU (mmol/l)	8.912±2.29	9.176±2.73
TG (mmol/l)	0.446±0.11	0.476±0.15
CHOL (mmol/l)	2.283±0.72	1.914±0.92
HDL-C (mmol/l)	0.792±0.08	0.714±0.10
LDL-C (mmol/l)	0.951±0.58	0.832±0.70

**Table 3:** The routine blood examination pre- and post-GO double layer drug coating stent implantation (*n*>3)

Project (unit)	Pre-stent implantation	Post-stent implantation
RBC (10^12^/l)	5.653±0.48	5.488±0.68
HGB (g/l)	122.7±11.04	114±13.88
MCV (fl)	67.02±2.23	67.93±2.10
MCH (pg)	21.71±0.83	21±2.82
MCHC (g/l)	323.9±6.06	308.6±37.09
RDW-CV (%)	32.2±1.77	62.47±95.49
HCT (%)	0.374±0.04	0.371±0.04
WBC (10^9^/l)	4.94±0.94	4.88±1.61
PLT (10^9^/l)	359±111.27	301.5±131.57
MPV (fl)	6.99±0.72	6.04±2.19
P-LCR (%)	6.29±3.00	4.29±2.50
PDW (fl)	7.87±1.17	6.7±2.47

### *In vivo* stent implantation and animal experiment of GCC stents

**Figure 7:**
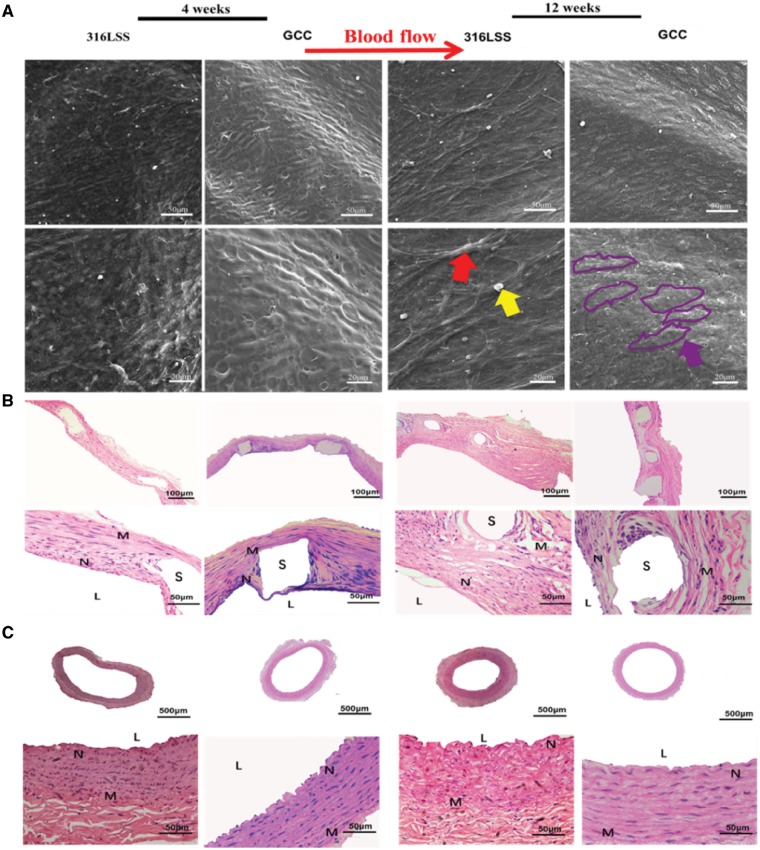
The Endothelialization and restenosis of GCC stents. (**A**) Complete neointima is generated at 4 weeks after implanting 316L SS and GCC stents. Blood components (yellow arrow) adhered and fibrous tissue-like materials (red arrow) appeared on the surface of 316L SS stents. The endothelial cells in GCC exhibited cobblestone-like (purple coils) re-arrangement following the direction of blood flow (purple arrow) at 4 and 12 weeks after stent implantation, respectively. (**B**) GCC inhibits intimal hyperplasia and the generation of neointima (S, stent; N, neointima; L, lumen; M, media). (**C**) The cells in intima and media are disordered and elastic plate is not clear or lacking at the distal and proximal sites after implanting 316L SS but not GCC stents

Animal experiments were further conducted to characterize the properties of stents after various coatings. The progress of re-endothelialization was examined by SEM after implanting the 316L SS and GCC stents for 4 and 12 weeks, respectively. The results show that re-endothelialization in both groups was completed at 4 weeks, and the surfaces were covered partially with cobblestone-like endothelial cells (Fig. 7A). There were blood components adhered to the endothelium on the 316L SS stents after 4 weeks implantation and fibrous tissue-like materials appeared after 12 weeks. However, nearly no platelets and fibrin were found on GCC stents after 12-week implantation.

To evaluate the effects of DTX released from GCC stents *in vivo*, the histo-morphometric analysis was performed after implantation for 4 and 12 weeks, respectively (Fig. 7B). The data show that after implanting 316L SS stents for 12 weeks, there were considerable levels of neointima hyperplasia and angiogenesis. For GCC stents, there were relatively low levels of neointima hyperplasia and its thickness was uniform after 4- and 12-week stent implantation. Compared with 316L SS stents, there were much lower levels of hyperplasia in GCC stents, suggesting that DTX and Hep may play important roles in inhibiting VSMC proliferation. Furthermore, the cells in the intima and media at the sites of stent implantation for 316L SS stents were disordered and the elastic plate was not clear even lacking (Fig. 7C). However, vascular endothelial cells and VSMCs in the intima and tube wall for GCC group were normal, mature, and complete and the elastic plate was parallel, wavy, and clearly visible.

## Discussion

The implantation of DES has a wide range of applications in clinical treatment and has become an effective method for treating cardiovascular diseases [26]. 316L SS is a backbone material of medical cardiovascular stents due to its high ductility, high mechanical strength and good fatigue properties [27, 28]. The preparation of stent coating has important influence on the physical, chemical and biological properties. In this study, 316L SS stents were modified with GO/DTX and CMC/Hep double-layer coating. Self-polymerization reaction could occur in DA in alkaline solution and the generated polydopamine was firmly adhered on the surface, which was suitable for subsequent modification. The surfaces coated with DAC, GDC or GCC were uniform without aggregation and particle deposition. The surface roughness was <50 nm and would not affect the biocompatibility in blood [29, 30]. Compared with many other coating methods, the influence of the coatings in this study on surface roughness was minimal, which highlights the superiority of ultrasonic spraying. Water contact angle reflects the hydrophobicity *in vivo*. The hydrophilic coating adsorbs albumin not platelets but adsorbs globulin, leading to platelet adhesion and activation of thrombosis. The water contact angle increased after coating with DAC or GDC, probably due to the strong hydrophobic interactions of benzene ring structure and DTX with DA molecules. The GCC coating had the best hydrophilicity, which might be due to the large number of hydrophilic groups in Hep and related to the ability of anti-globulin and anti-platelet adhesion.

GO as an oxidative derivative of graphene has been widely used in the biomedical field because of its large specific surface area and good adsorption performance [31]. However, GO often exhibits toxicity to some extent and side effects at certain concentrations [32]. GO could also induce apoptosis and cell cycle arrest through extracellular regulated protein kinases pathway and autophagy via calcium-dependent phosphorylation of c-Jun N-terminal kinases [33, 34]. Liao *et al*. [35] found that GO of different sizes exhibits different levels of toxicity.

It is necessary to modify the GO coating as to keep its own advantages and have better biosafety at the same time. The aqueous soluble GO-copper nanocomposites coating enhances the adhesion and osteogenic differentiation of rat bone marrow stem cells [36], heparin-loaded GO coating on titanium surface improved the blood compatibility and endothelial cell behaviors [37]. In this research, we prepared the coating using ultrasonic atomization spraying which has been proved to get high quality coatings from our previous studies [15, 16, 38] and the designed double-layer coating greatly increased the biocompatibility of GO and implemented the multifunction of the coating with loaded multiple drugs.

The blood compatibility characteristic is critical for the stents in the treatment of cardiovascular diseases. The hemolysis of all coatings in this study was <5%, which met the safety requirement according to the national standards. The hemolytic rate of GCC was the lowest among the coatings of 316L SS, DAC and GDC. After the implantation, the stent interacts with blood cells, including platelets [22]. The poor blood compatibility of coating may lead to not only hemolysis but also the formation of thrombosis [39, 40]. In this study, the number of the adhered platelets in GCC was the lowest compared with 316L SS, DAC and GDC, showing good anti-thrombotic ability. Furthermore, the platelet morphology index of the adhered platelets in GCC was the smallest, suggesting that GCC has the minimum effect on platelet morphology and activation. This may be due to Hep eluted from the coating surface, since it is known that Hep is an anti-thrombotic drug and has anti-coagulant and anti-platelet activation and aggregation functions [40, 41].

Cell proliferation tests show that the coating with DTX significantly inhibits the proliferation and activity of VSMCs, which is consistent with the findings that DTX can inhibit the proliferation and migration of SMCs [18]. DTX, a derivative of paclitaxel (taxol), has been widely used as an anti-neoplastic drug in the treatment of a variety of solid cancers, such as breast cancer, gastric cancer, hormone-refractory prostate cancer, head and neck cancer, and non-small-cell lung cancer [42, 43]. In addition, the OD value of GCC was slightly but not significantly less than GDC, suggesting that Hep may also play a role in inhibiting VSMC proliferation although the effect is not as good as DTX [44, 45]. Heparin has been widely used as an anticoagulant and can suppress intimal hyperplasia and inflammation and promote endothelium regeneration in some cases [46]. Hep can also inhibit the migration and proliferation of blood vessel cells, especially SMCs, through the effects on PKC-α expression [47]. Furthermore, GDC and GCC not only inhibit the proliferation of VSMCs, but also influence their morphology. VSMCs in GDC and GCC groups have prominent nucleus and remodeled cytoskeletal structure, probably due to the effects of DTX on microtubule [43].

Considering the toxicity of GO mentioned earlier, it is indispensable to test the biological safety of various coatings before implantation of GCC coated stent. Zebrafish is a typical animal model for toxicology studies and widely used in medical and environmental research [48, 49]. In this study, the GCC had no significant effects on the survival rate, hatching rate and heart rate of zebrafish embryos compared with the control group. The tested physiological indexes in all groups were in the normal ranges. These findings suggest that the GO coating does not show detectable toxicity to the development of zebrafish embryo and larvae. This may be due to the facts that the amount of GO in various coatings is low and that the covalent interactions of the amino groups in DA with the carboxyl groups in GO may decrease the GO toxicity [50].

Furthermore, the GCC stents have been implanted into rabbit carotid artery to study the effects on endothelial repair, restenosis and anti-thrombosis. Compared with 316L SS stents, no ISR and thrombosis and significant intimal hyperplasia were observed in the GCC stents group, which further confirms that DTX and Hep play an important role in inhibiting VSMC proliferation. The rate of vascular restenosis was ∼20–40% after implanting bare metal stents, because the damage to the endothelium after 316L SS stent implantation is unable to be recovered, leading to the occurrence of VSMC proliferation, phenotypic transformation and extracellular matrix secretion [51–54]. DAC stents and GDC stents are an intermediate state of GCC stents. Besides, the GCC stents have no significance effects on the main organs of rabbits. And the animal data further demonstrate that the GCC stents have the excellent effects of anti-proliferation and anti-thrombosis.

## Conclusion

This study has demonstrated that the GO double-layer drug coated stents are robust without the shedding problem. The stents with various coatings have good blood compatibility and can effectively inhibit the proliferation of VSMCs. GO double-layer drug coating significantly suppresses the adhesion and activation of platelets and the proliferation and migration of VSMCs. The *in vivo* rabbit study has demonstrated GO coating inhibits intimal hyperplasia and thrombosis and promotes endothelialization. The toxicity tests suggest that the coating has no significant effect on the main physiological indicators such as survival rate, hatching rate and heart rate of zebrafish embryos. GO double-layer drug coating stents do not lead to abnormal development of zebrafish embryos and juvenile fish.

## Funding

This work was supported by grants from the National Key Research and Development Program of China (2016YFC1102305), the Fundamental Research Funds for the Central Universities (2018CDPTCG0001-10), and the support from the Chongqing Engineering Laboratory in Vascular Implants and the Public Experiment Center of State Bioindustrial Base (Chongqing).

*Conflict of interest statement*. None declared.
